# Whole-Genome Sequences of Chlamydia psittaci Strain HJ, Isolated from Meat Pigeons with Severe Respiratory Distress and High Mortality

**DOI:** 10.1128/genomeA.00035-15

**Published:** 2015-03-05

**Authors:** Qiang Zhang, Zongxue Wu, Ruixue Sun, Jun Chu, Er Han, Yinxin Zhang, Yong Ling, Yanping Gong, Dongfang Li, Honglong Wu, Cheng He, Peixiang Ni

**Affiliations:** a Key Laboratory of Animal Epidemiology and Zoonosis of Ministry of Agriculture, College of Veterinary Medicine, China Agriculture University, Beijing, China; b BGI-Tianjin, Tianjin, China; c Tianjin Translational Genomics Center, BGI-Tianjin, Tianjin, China

## Abstract

The obligate intracellular Gram-negative bacterium Chlamydia psittaci causes systemic disease in psittacine birds, domestic poultry, and wild fowl. Importantly, C. psittaci may cause pneumonia, encephalitis, endocarditis, and even death in humans. The potential of pigeons as a source of human psittacosis is supported worldwide by relatively high seroconversion rates in the birds. This study reports the whole-genome sequencing of C. psittaci strain HJ, isolated from meat pigeons suffering from severe pneumonia and high mortality in 2013 in Hebei, China.

## GENOME ANNOUNCEMENT

Chlamydiosis is an avian disease caused by the bacterium Chlamydia psittaci. Infected birds suffer from pneumonia, poor growth, diarrhea, and central nervous system disturbances that depend on the chlamydial genotype involved and the affected bird species (1). Columbiform birds have been ranked as the second major reservoir of C. psittaci (2), which is also known to be transmissible to humans, causing a potentially severe zoonotic infection (1, 3). The prevalence of C. psittaci in meat pigeons and the associated risk to humans have been documented (4).

Here, we report the genome sequence of C. psittaci strain HJ, which was isolated in China from the lungs of a 28-day-old silver king pigeon that had severe respiratory distress symptoms for 20 days (4). C. psittaci isolate HJ was determined to be a virulent strain in specific-pathogen-free (SPF) chickens when compared with virulent C. psittaci 6BC and mild C. psittaci CB7 (5).

The lung tissues were collected from diseased meat pigeons during the clinical phase. After treatment with gentamicin (200 µg/ml) and vancomycin (1 mg/ml) in the lung tissue suspension for 30 min, the supernatant preparations were inoculated onto a BGM cell monolayer for three passages. The chlamydial antigens were detected using direct immunofluorescence test kits (Oxoid Ltd., United Kingdom) and the amount of inclusion-forming units (IFU) of C. psittaci strain HJ was determined to be 10^8.5^ IFU per ml, as previously described (6). The purification of chlamydial elementary body (EB) by meglumine gradient and chlamydial genomic DNA preparations from purified EBs were performed as previously described (5).

This genome was sequenced with Illumina sequencing technology after a 500-bp DNA library was constructed to determine the complete genomic sequence of the strain HJ, while 10 million paired-end reads were obtained with read lengths of 100 bp. Almost 900× depth was achieved for the C. psittaci 6BC genome (7).

The reads from the host cell were filtered by aligning the raw data to the genome of Chlorocebus sabaeus with SOAPaligner (version 2.21). *De novo* assembly was performed using SOAP*denovo* (version 1.05). The genome of HJ was found to be 1,237,960 bp in size, and 81 scaffolds with an *N*_50_ size of 95,161 bp were assembled. The G+C content of strain HJ is 39.02%, which is similar to that of other *Chlamydiaceae* (8, 9). Three scaffolds with a length of 7,972 bp were identified as plasmid sequence when compared with the sequence of the C. psittaci 6BC strain.

The annotation of this genome was performed by an in-house pipeline, and 1,327 putative coding sequences of the chromosome of HJ were predicted using the Glimmer3 software package. Nine putative coding sequences were identified in the plasmid sequence of the HJ strain. This strain has one rRNA operon and 37 tRNAs, as determined by RNAmmer and tRNAscan-SE, respectively (10, 11). The molecular characterization of this isolate by the construction of a phylogenetic tree with single nucleotide polymorphisms based on those released public genome sequences revealed that it belongs to genotype B.

### Nucleotide sequence accession numbers.

This whole-genome shotgun project of strain HJ has been deposited at DDBJ/EMBL/GenBank under the accession no. JPIH00000000, and the version cited in this paper is JPIH01000000.
